# Facile Synthesis of Highly Emissive All-Inorganic Manganese Bromide Compounds with Perovskite-Related Structures for White LEDs

**DOI:** 10.3390/molecules27238259

**Published:** 2022-11-26

**Authors:** Ping Gao, Suwen Cheng, Jiaxin Liu, Junjie Li, Yanyan Guo, Zhengtao Deng, Tianshi Qin, Aifei Wang

**Affiliations:** 1 Key Laboratory of Flexible Electronics (KLOFE) & Institute of Advanced Materials (IAM), Jiangsu National Synergetic Innovation Center for Advanced Materials (SICAM), Nanjing Tech University (Nanjing Tech), Nanjing 211816, China; 2 State Key Laboratory of Analytical Chemistry for Life Science, National Laboratory of Micro-Structures, College of Engineering and Applied Sciences, Nanjing University, Nanjing 210023, China

**Keywords:** perovskite, Cs_3_MnBr_5_, manganese halide, phosphor, white LEDs

## Abstract

Lead-free all-inorganic halide materials with different Mn^2+^-based crystal structures (Cs_3_MnBr_5_ and CsMnBr_3_) were obtained using a convenient synthetic method. Cs_3_MnBr_5_ had a bright green emission (522 nm), with a unique single-exponential lifetime (τ_avg_ = 236 µs) and a high photoluminescence quantum yield (82 ± 5%). A red emission was observed in the case of the CsMnBr_3_ structure with a two-exponential fluorescence decay curve, and the lifetime was 1.418 µs (93%) and 18.328 µs (7%), respectively. By a judicious tuning of the synthetic conditions, a mixed phase of Cs_3_MnBr_5_/CsMnBr_3_ was also produced that emitted white light, covering almost the entire visible spectrum. White-light-emitting diodes (WLEDs) with color coordinates (0.4269, 0.4955), a color temperature of (3773 K), and a color rendering index (68) were then fabricated using the as-prepared powder of mixed phases of Cs_3_MnBr_5_/CsMnBr_3_ with a commercial UV LED chip (365 nm).

## 1. Introduction

White LEDs garner a great deal of attention, and their commercialization has expanded over the years for high-volume applications, including general lighting and backlight displays [1,2,3,4,5,6]. In reality, the desirable properties of LEDs, such as their high efficiency, slimmer profile, fast switching time, and low contamination, make them valuable for use in modern liquid-crystal display (LCD)-based devices compared to a conventional cold cathode fluorescent lamp (CCFL) [7,8,9,10]. Traditional phosphor materials used in LEDs are mainly composed of nitride-based phosphors and rare-earth-metal-doped oxides, their synthesis always requiring high-temperature annealing and the use of costly rare earth metals, which, in turn, severely increases the commercialization costs [11,12,13,14]. In view of this, organic or inorganic–organic hybrid phosphor materials were then used in an attempt to reduce the reliance on rare earth metals, but their relatively low thermal and chemical stability limit their practical applications [15,16]. In addition, white LEDs are normally composed of two or more different phosphor to broaden the emission band, which always involved complicated synthetic techniques. Given the above-mentioned issues, the development of materials that feature brilliant backlighting in high-gamut displays, are easy to prepare, have a low cost, have high photoluminescence quantum yields (PLQYs), and have a brilliant thermal stability is highly desirable.

Recently, metal halide perovskites have gained enormous attention as luminescent materials for lighting and displays, owing to their high quantum yield, color tunability, and narrow emission features [17,18,19,20,21]. Despite these fascinating characteristics, nearly all high-performance perovskites contain heavy metal lead, which poses a great threat to the ecological environment and human health. It is of great interest to search for ecofriendly alternatives through the partial or complete replacement of lead. Transition metal ion doping is considered as a viable solution, owing to the fact that the transition metal ligand-field excited states usually reside within the band gap of the host semiconductors [22,23,24,25]. In particular, the doping of halide perovskites with Mn^2+^ has been widely studied because it potentially imparts novel optical, electronic, and magnetic functionalities. This objective has been recently achieved in the case of organic halide perovskite, wherein 90% of lead was replaced, simultaneously retaining its unique optoelectronic properties of excitonic and Mn^2+^-associated emissions [26,27,28].

**Figure 1:**
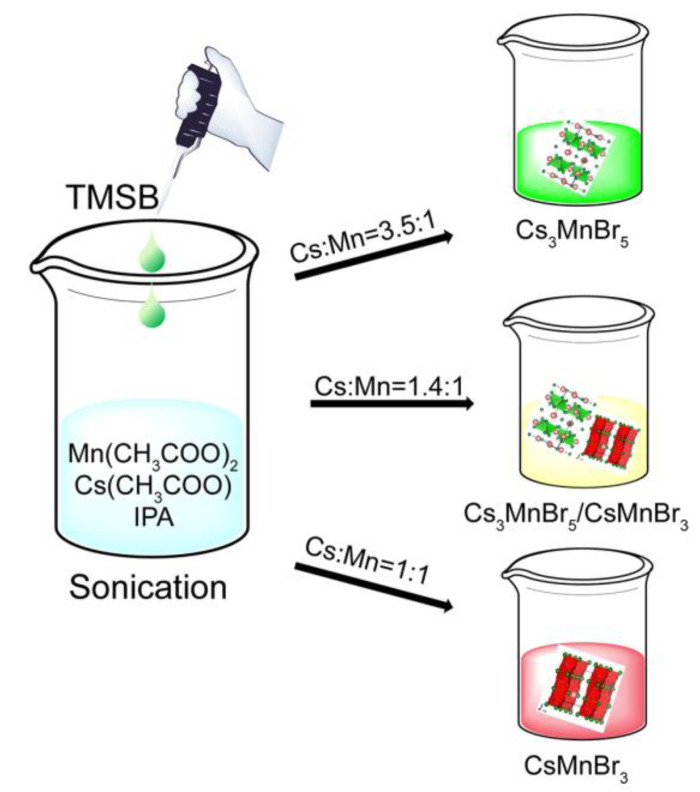
Schematic illustration of the synthetic procedure for Cs_3_MnBr_5_ and CsMnBr_3_ using a room-temperature sonication crystallization strategy.

Recent investigations have also shown that many Mn(II) compounds, especially inorganic–organic hybrid materials, often exhibit excellent optical properties (emit strong tunable fluorescence). The high fluorescence quantum yields and convenient and economical fabrication protocols make them potential candidates as emitting materials. Xiong and coworkers reported a hexagonal stacking manganese halide perovskite of (pyrrolidinium) MnCl_3_ that exhibited an intense red luminescence, and tetrahedrally coordinated [N-methylpyrrolid inium] _2_MnBr_4_ showed an intense green emission [29,30]. In another effort, the Deng group prepared a pyridine manganese halide perovskite (C_5_H_6_N)_2_MnBr_4_ with a strong green light emission and C_5_H_6_NMnCl_3_ with a red emission, and used the resulting materials for the fabrication of white-light-emitting diodes (WLEDs) [31]. In the previous reports, the inherent instability obstacle of the materials due to the organic component still need to be solved. Herein, we presented a facile synthetic approach used to produce all inorganic manganese-based perovskites (Cs_x_MnBr_x+2_). We obtained Cs_3_MnBr_5_ and CsMnBr_3_ with varied structures by controlling the Cs/Mn ratio. The as-fabricated Cs_3_MnBr_5_ showed a d-d transition of Mn ions at 522 nm, whereas the CsMnBr_3_ produced an emission band at 655 nm corresponding to Mn clusters. A manganese halide compound with a Cs/Mn ratio of 1.4:1 generated a photon emission covering almost the entire visible spectrum. Cs_3_MnBr_5_ and CsMnBr_3_ were successfully synthesized by a one-pot sonication method at room temperature, and the detailed synthetic procedure is schematically illustrated in Figure 1.

## 2. Results and Discussion

### 2.1. The Structure Morphology and Compositional Analysis of Cs_x_MnBr_x+2_

**Figure 2:**
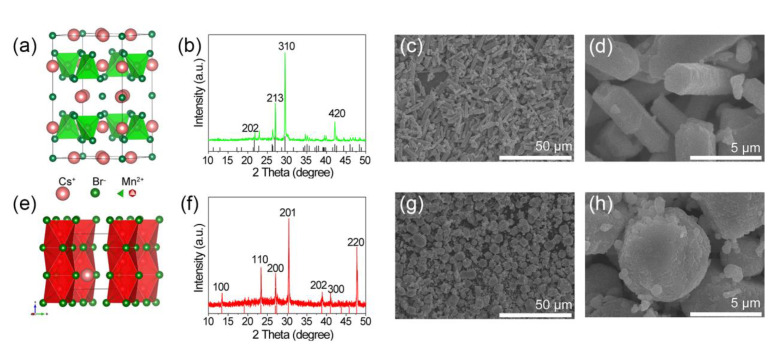
(**a**,**e**) Crystal structure of Cs_3_MnBr_5_ (tetragonal, space group I4/mcm, a = 9.596 Å) and CsMnBr_3_ (hexagonal, space group P 63/mmc, a = 7.618 Å); (**b**,**f**) XRD patterns of Cs_3_MnBr_5_ and CsMnBr_3_ (black) measured under ambient conditions, where the red columns at bottom are the standard patterns related to tetragonal Cs_3_MnBr_5_ and hexagonal CsMnBr_3_; low- and high-resolution SEM images of (**c**,**d**) Cs_3_MnBr_5_ and (**g**,**h**) CsMnBr_3_.

The crystal structures of Cs_3_MnBr_5_ and CsMnBr_3_ are shown in Figure 2a,b,e,f, the crystal structures of the as-prepared samples were confirmed by powder X-ray diffraction (XRD). XRD data indicated that the crystal structure of Cs_3_MnBr_5_ was tetragonal (I 4/mcm) with lattice parameters a = b = 9.596 Å, c = 15.57 Å, and the obvious diffraction peaks at 2θ = 21.8, 22.9, 26.3, 27.0, 29.5, and 42.1° correspond to diffractions from the {202}, {004}, {114}, {213}, {310}, and {420} planes (Figure 2b). CsMnBr_3_ crystallized with a hexagonal structure (P63/mmc) with a = b = 7.618 Å, c = 6.519 Å, and the obvious diffraction peaks at 2θ = 13.4, 23.4, 27.0, 30.4, and 47.7 corresponded to diffractions from the {100}, {110}, {200}, {201}, and {220} planes (Figure 2f). No detectable impurity was observed in the above samples. In Figure 2c,d, scanning electron microscopy (SEM) images showed rod-like crystals of Cs_3_MnBr_5_ with a diameter of 1μm and length of 10 μm, and CsMnBr_3_ (Figure 2g–f) was observed with a sphere shape with average sizes of 500 nm. The large size of the two crystals also indicated a good degree of crystallinity. EDS mapping suggested (Figure S1), in Cs_3_MnBr_5_, Cs, Mn, and Br, an atom ratio of 3.9:1:5.6, and in CsMnBr_3_, Cs, Mn, and Br, an atom ratio of 1:1:2.8.

### 2.2. Optical Properties of Cs_3_MnBr_5_ and CsMnBr_3_

**Figure 3:**
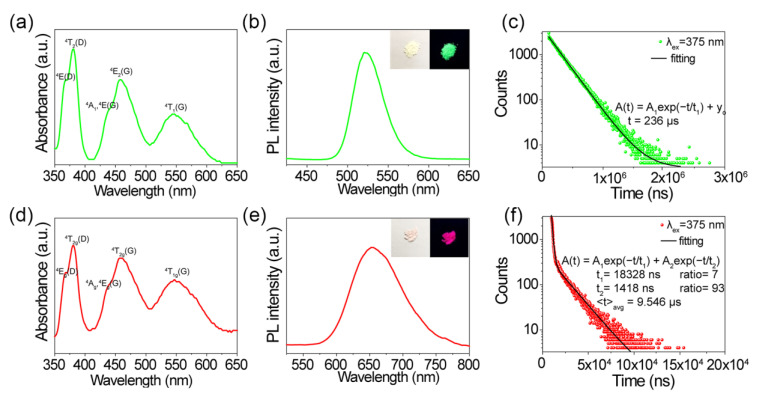
Absorption and corresponding emission spectra of Cs_3_MnBr_5_ (**a**,**b**) and CsMnBr_3_ (**d**,**e**) at ambient conditions. Inset: photographs of Cs_3_MnBr_5_ and CsMnBr_3_ under room and UV light (385 nm). Time-resolved PL decays and fitting curves at 522 nm in Cs_3_MnBr_5_ (**c**) and 655 nm in CsMnBr_3_ (**f**), and the excitation wavelength was 375 nm.

Figure 3a,b,d,e show the absorption and associated emission spectra of Cs_3_MnBr_5_ and CsMnBr_3_ at ambient conditions. The absorption peaks ranging from the visible to UV region correspond to an electronic transition from the ^6^A_1_ ground state of Mn^2+^ to different excited states of MnBr_χ_ in Cs_3_MnBr_5_ and CsMnBr_3_. In the absorption spectra of Cs_3_MnBr_5_, the intense absorption bands at 368, 380, 442, 458, and 544 nm belong to ^6^A_1_→^4^E(D), ^6^A_1_→^4^T_2_(D), ^6^A_1_→^4^A_1_,^4^E(G), ^6^A_1_→^4^T_2_(G), and ^6^A_1_→^4^T_1_(G), respectively (Figure 3a). Similarly, in the case of the absorption of CsMnBr_3_, intense absorption bands associated with ^6^A_1_→^4^E_g_(D) (368 nm), ^6^A_1_→^4^T_2g_(D) (381 nm), ^6^A_1_→^4^A_g_,^4^E_g_(G) (437 nm), ^6^A_1_→^4^T_2g_(G) (458 nm), and ^6^A_1_→^4^T_1g_(G) (544 nm), respectively, were observed (Figure 3d). Correspondingly, Cs_3_MnBr_5_ exhibited a green emission (522 nm) (Figure 3b) with very high photoluminescence quantum efficiencies (PLQYs) of 82 ± 5%, the PL peak position of CsMnBr_3_ was at 655 nm (Figure 3e), and PLQYs were as low as 11%. The emission at 522 and 655 nm could be ascribed to the ^4^T_1_→^6^A_1_ transition of the tetrahedrally coordinated Mn^2+^ ion from the [MnBr_4_]^2−^ anion and the ^4^T_1g_→^6^A_1_ transition of the octahedrally coordinated Mn^2+^ ion from the [MnBr_6_]^4−^ chain, respectively [32]. Obviously, it is important to understand the distinguishable differences in the PL spectra of these two compounds with similar absorption profiles. As they have the same Cs site, the MnBr_χ_ cluster separation could actually play an important role in influencing their optical transition. As reported, compounds with Mn^2+^ in a tetrahedral environment usually emit in the green region, whereas those with octahedral coordinated Mn^2+^ ions tend to have orange to red emissions [33]. Mn^2+^ is fourfold coordinated by Br^−^ to form a [MnBr_4_]^2−^ tetrahedron in Cs_3_MnBr_5_, while the Mn-Mn distance (3.26 Å) in CsMnBr_3_ forms linear chains of a face-sharing [MnBr_6_]^4−^ octahedron [34]. The latter is conducive to the formation of a linear Mn–Mn chain, and the interchain Mn–Mn interaction is several orders of magnitude larger than that of Cs_3_MnBr_5_, which is almost negligible due to the larger Mn–Mn distance of 6.785 Å. In the case of magnetic coupling between neighbouring manganese ions in CsMnBr_3_, their d–d emission band likely shows a red shift [35,36,37]. Time-resolved PL decays and fitting curves of Cs_3_MnBr_5_ and CsMnBr_3_ are shown in Figure 3c,f. A longer lifetime of 236 µs was found in Cs_3_MnBr_5_, which is consistent with the long durations of the self-trapped excited state present in the Mn complex as reported in the literature [38]. The time-resolved PL curve fit well to the single exponential decay function, which suggested that the PL decay route is related to little-to-no non-radiative processes, consistent with the relatively higher PLQY achieved in Cs_3_MnBr_5_. However, the time-resolved PL decay of CsMnBr_3_ was fitted to a two-exponential decay equation, and the average lifetime of PL decay was found to be 9.546 µs. After two-exponential fitting, we estimated the PL decay for CsMnBr_3_ and had two components, 1.418 µs (93%) and 18.328 µs (7%); the former originates from the surface-state recombination, and the latter can be attributed to the intrinsic recombination. Taken together, the fluorescence quantum yield of CsMnBr_3_ was found to be lower than that of Cs_3_MnBr_5_.

### 2.3. Controllable Photoluminescence and Crystal Structures of Cs_x_MnBr_x+2_

**Figure 4:**
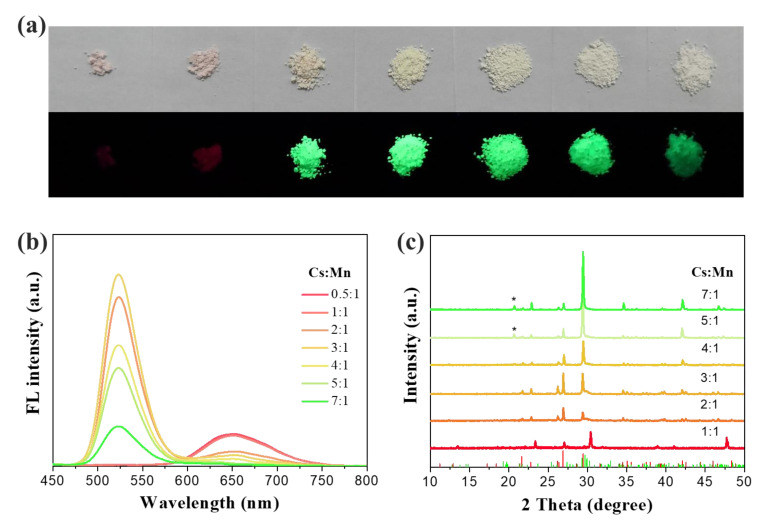
Photographs (**a**) and PL emission spectra (**b**) of the as-prepared Cs_x_MnBr_x+2_ samples with different Cs/Mn ratios; (**c**) the XRD patterns of Cs_x_MnBr_x+2_ samples with different Cs/Mn ratios.

Figure 4a shows the images of the as-prepared Cs_x_MnBr_x+2_ samples with different Cs/Mn ratios under ambient light and UV lamp irradiation (365 nm). The relationship between the intensity/position of the dominant emission and Cs/Mn ratios can be evidently seen (Figure 4b). It is clearly noticed that the green emission band appears and gradually suppresses the red emission with increasing Cs/Mn ratios. When the nominal Cs/Mn ratios is 0.5 or 1, there is only one red emission peak (655 nm) corresponding to hexagonal CsMnBr_3_; however, with increasing Cs/Mn ratios, the Cs_x_MnBr_x+2_ samples underwent a phase transition (Figure 4c). When the Cs/Mn ratios increased from 2 to 3.5, the host diffraction peaks of tetragonal Cs_3_MnBr_5_ experienced an increase, and the additional orthorhombic phase of the sample (Cs_2_MnBr_4_) gradually decreased and eventually disappeared. Correspondingly, the intensity of the green emission increased, whereas the red emission intensity dramatically reduced. When the nominal Cs/Mn ratios increased to 3.5 or greater, the sample transformed into a CsBr phase in addition to tetragonal Cs_3_MnBr_5_ due to the excessive presence of Cs in the precursors. Notably, only one green emission peak (522 nm) remained in the sample, whose intensity gradually decreased as the Cs/Mn ratios increased, and the red emission was gradually suppressed to an almost undetectable level.

### 2.4. Application in UV Pumped White LEDs

**Figure 5:**
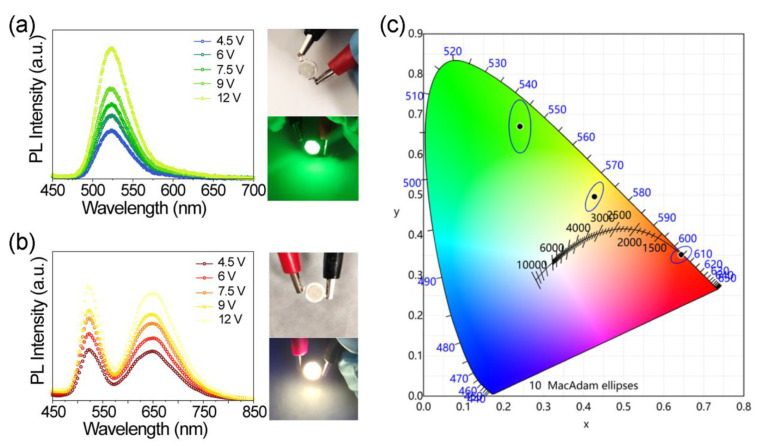
Emission spectrum of the constructed green LED (**a**) and warm white LED (**b**) at different driving voltages. Inset: photographs of the as-fabricated UV (365 nm) pumped LED devices with and without power input. (**c**) Chromaticity coordinates of different Cs/Mn ratios plotted on CIE1931 chromaticity chart: 3.5:1 (triangle), 1.4:1 (star), 1:1 (round).

As mentioned before, we can tune different Cs/Mn feed ratios to obtain various emission wavelengths. We fabricated two types of light-emitting diode (LED) devices using pure Cs_3_MnBr_5_ (Cs/Mn = 3.5:1) and the mixture of Cs_3_MnBr_5_ and CsMnBr_3_ (Cs/Mn = 1.4:1) as light emitters. Figure 5a,b show the color stability of the as-fabricated LEDs at different driving voltages. The light intensity curves increase steadily without distortion when the voltage increases from 4.5 V to 12 V. Taken together, we envision Cs_X_MnBr_X+2_ as potential green and near-warm white phosphors for display backlight applications. The color coordinates of the former were calculated to be (0.2407, 0.6699) (Figure 5c), implying that this phosphor can be used for green LED applications. The inset in Figure 5a shows the digital photograph of the as-fabricated UV (365 nm) pumped LED devices with a powder input, and an intense green emission can be observed. In a similar situation, the color coordinates of the latter were calculated to be (0.4269, 0.4955) (Figure 5c). As observed in the inset of Figure 5a,b, the constructed LED shows a strong green and near-warm white emission, indicating that this phosphor can be used for green and warm white LED application.

### 2.5. Magnetic and Thermostability

**Figure 6:**
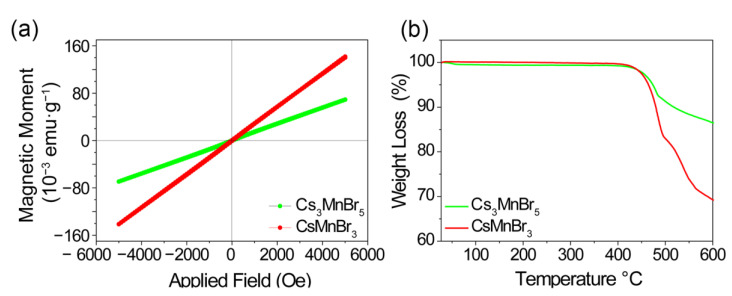
(**a**) Magnetic properties of Cs_3_MnBr_5_ and CsMnBr_3_ at room temperature. (**b**) Thermogravimetric (TGA) curves of Cs_3_MnBr_5_ and CsMnBr_3_ in air atmosphere at a heating rate of 10 °C/min.

Regarding the investigation of the Mn−Mn coupling effect on luminescence in our sample, Figure 6a shows M–H curves of Cs_3_MnBr_5_ and CsMnBr_3_ obtained by a vibrating sample magnetometer (VSM) at room temperature. Both of them show clearly paramagnetic behavior, which demonstrates that the red emission band was caused by the ferromagnetic coupling of Mn-Mn in CsMnBr_3_, and not by the crystal field and crystal structure [39]. In order to investigate the thermal stability of Cs_3_MnBr_5_ and CsMnBr_3_, thermo-gravimetric analysis (TGA) was carried out from room temperature to 600 °C. As displayed in Figure 6b, both of them have a perfect thermal stability up to T = 425 °C.

## 3. Materials and Methods

### 3.1. Chemicals

Cesium acetate (CH_3_COOCs, 99%), manganese(II) acetate Mn (CH_3_CO_2_)_2_, AR, 99%, trimethylbromosilane (TMBS, 98 wt% in water), and isopropanol (IPA, 98 %)) were used. All chemicals were purchased from China Shanghai Aladdin Reagent Company.

### 3.2. Synthesis of Cs_3_MnBr_5_ and CsMnBr_3_

For the typical synthesis of rod-like crystals, Cs_3_MnBr_5_, Mn(CH_3_COO)_2_ (0.4 mmol) and CH_3_COOCs (1.4 mmol) used as manganese and cesium sources, respectively, were dissolved in isopropanol (10 mL) by sonication until all Mn (CH_3_COO)_2_ and CH_3_COOCs salt was dissolved. Then, TMBS used as the bromide source (4 mmol) was added to the above precursor solution and a whiteish precipitation was formed immediately under strong sonication. After another 30 min sonication, the reaction mixture was filtered and a white product was obtained. 

The synthetic process for CsMnBr_3_ was identical to the one mentioned above, but the molar concentrations of Mn (CH_3_COO)_2_ and CH_3_COOCs were changed to a stoichiometric ratio of 1:1.

White-light-emitting samples of Cs_3_MnBr_5_/CsMnBr_3_ mixed phases were obtained by tuning the Cs/Mn precursor ratio to 1:4:1.

### 3.3. LEDs Lamp Fabrication

Various as-synthesized phosphor powders were blended well with 25% PS dichloromethane solution. The blended phosphors of PS paste were dropped on top of the UV LED chips (365 nm) and dried in air to form LED lamp.

### 3.4. Characterization

Powder X-ray diffraction (PXRD): PXRD was measured with a Bruker AXS D8 X-ray diffractometer equipped with monochromatized Cu Kα radiation (λ = 1.5418 Å). The diffraction pattern was scanned over the angular range of 5–50° (2θ) with a step size of 0.01 at room temperature. Scanning electron microscopy (SEM): SEM was performed on a Japan Hitachi (S-3400N) operating at 20 kV, equipped with energy-dispersive X-ray spectroscopy (EDS) detector. Ultraviolet and visible (UV–vis) absorption spectroscopy for solid samples: UV–vis spectra were recorded with a Shimadzu UV-3600 plus spectrophotometer equipped with an integrating sphere under ambient conditions. Photoluminescence (PL) spectra were obtained with a Horiba PTI QuantaMaster 400 steady-state fluorescence system. Absolute photoluminescence quantum yield (PLQY) measurements for solid samples: The absolute fluorescence quantum yields were measured using a Horiba PTI QuantaMaster 400 steady-state fluorescence system with an integrated sphere and double-checked with a Hamamatsu Photonics Quantaurus-QY (model: C11347-11) under ambient conditions. Time-resolved photoluminescence lifetime measurements for solid samples: Time-resolved PL emission decay curves were collected at room temperature and detected by a Nikon Ni-U Microfluorescence Lifetime System (Confotec MR200, SOL, Belarus) with a 375 nm picosecond laser.

## 4. Conclusions

In this work, we presented a rapid and mild synthetic protocol for the synthesis of Mn(II) halide-based all-inorganic lead-free perovskite with a high PLQY. By a simple tuning of the feed ratios, two different crystals structures (Cs_3_MnBr_5_ and CsMnBr_3_) with tunable fluorescence characteristics were obtained. The as-prepared Cs_3_MnBr_5_ displayed a green emission, with the highest PLQY of up to 82 ± 5% and with a long lifetime of 236 μs. When the feeding ratio of Cs/Mn was tuned to 1.4:1, the resulting mixture of Cs_3_MnBr_5_ and CsMnBr_3_ emitted a white light covering almost the entire visible spectrum. Based on the high optical quality of the products, UV pumped green and warm white LEDs were fabricated using the as-prepared Cs_3_MnBr_5_ and a mixture of Cs_3_MnBr_5_/CsMnBr_3_ as light emitters. Overall, as an intriguing prototype, this work not only paves the way for the simple synthesis of highly emissive, low-cost, environmentally benign halide materials, but also implies a great potential for optoelectronic devices.

## Data Availability

Not applicable.

## Supplementary Materials

The following supporting information can be downloaded at: https://www.mdpi.com/article/10.3390/molecules27238259/s1, Figure S1: EDS spectrum.

## Sample Availability

Samples of the compounds are available from the authors.
