# Supplementary material for: Facile Synthesis of Highly Emissive All-Inorganic Manganese Bromide Compounds with Perovskite-Related Structures for White LEDs
Source: Molecules. 2022 Nov 26;27(23):8259. doi: 10.3390/molecules27238259 (PMC9736304; doi:10.3390/molecules27238259)
Supplement: Supplementary file 1 [file molecules-27-08259-s001.zip › molecules-2051432-supplementary.pdf]

Supporting Information

Facile Synthesis of Highly Emissive All-Inorganic Manganese Bromide Compounds with Perovskite-Related Structures for White LEDs

Ping Gao [1], Suwen Cheng [1], Jiaxin Liu [1], Junjie Li [1], Yanyan Guo [2] and Zhengtao Deng [2], Tianshi Qin [1] and Aifei Wang [1,*]

1 Key Laboratory of Flexible Electronics (KLOFE) & Institute of Advanced Materials (IAM), Jiangsu National Synergetic Innovation Center for Advanced Materials (SICAM), Nanjing Tech University (Nanjing Tech), Nanjing 211816, China
2 College of Engineering and Applied Sciences, State Key Laboratory of Analytical Chemistry for Life Science, National Laboratory of Micro-Structures, Nanjing University, Nanjing 210023, China
* Correspondence: iamafwang@njtech.edu.cn

Supplementary Figures

(a)
(b)

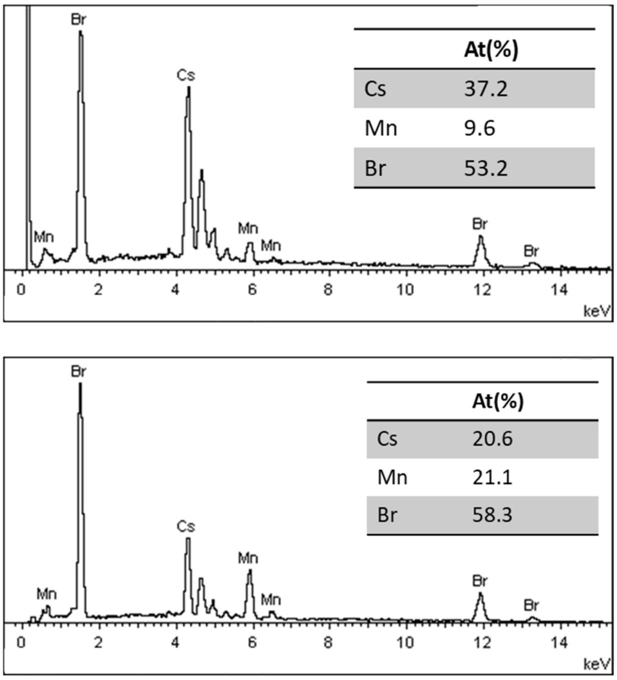

Figure S1. EDS spectrum of the (**a**) Cs_3MnBr_5 and (**b**) $CsMnBr_3$.
